# RECOGNIZING THE ROLE OF PETS IN HOME ASSESSMENTS FOR OLDER ADULTS

**DOI:** 10.1093/geroni/igad104.3555

**Published:** 2023-12-21

**Authors:** Carlyn Ellison Keeler, Linda Struckmeyer, Jennifer Applebaum

**Affiliations:** University of Illinois at Urbana-Champaign, Champaign, Illinois, United States; University of Florida, Gainesville, Florida, United States; University of Florida, Gainesville, Florida, United States

## Abstract

To support aging in place, health professionals may identify safety and accessibility concerns via home assessments. Although 50% of older adults share their homes with pets, standardized home assessments often overlook pet care, an instrumental activity of daily living (IADL). To bridge this gap in existing knowledge, we conducted four focus group sessions with older adult pet owners to identify potential elements for a novel assessment tool. We utilized directed content and thematic analysis, informed by the International Classification of Functioning, Disability and Health model. Researchers recruited a purposive sample of older adults (65 years or older) who were the primary caretakers of a dog or cat. Participants (N=30) discussed age-related changes to their body’s function that hindered their participation in pet care activities (e.g., the strength needed to pick up a pet). Participants explained how the interaction of their pets and housing features (e.g., stairs) contributed to falls and injuries. Further, personal factors (e.g., restricted income) may contribute to home safety issues (e.g., unable to afford needed modifications). The results indicate that pets’ behavior, care needs, and caregiving activities may impact home safety, thereby influencing the potential for aging in place. Findings support the development of holistic approaches when evaluating older adults’ functioning in their homes and emphasize the importance of pet care within home assessments.

